# Effect of a high dose atorvastatin as adjuvant therapy to mesalamine in attenuating inflammation and symptoms in patients with ulcerative colitis: a randomized controlled pilot study

**DOI:** 10.3389/fmed.2024.1490178

**Published:** 2025-01-22

**Authors:** Sumaiah J. Alarfaj, Sahar M. El-Haggar, Sahar K. Hegazy, Maha M. Maher, Monir M. Bahgat, Thanaa A. Elmasry, Sarah Alrubia, Amsha S. Alsegiani, Mostafa M. Bahaa

**Affiliations:** ^1^Department of Pharmacy Practice, College of Pharmacy, Princess Nourah Bint Abdulrahman University, Riyadh, Saudi Arabia; ^2^Clinical Pharmacy Department, Faculty of Pharmacy, Tanta University, El-Guiesh Street, El-Gharbia Government, Tanta, Egypt; ^3^Pharmacy Practice Department, Faculty of Pharmacy, Horus University, New Damietta, Egypt; ^4^Internal Medicine Department, Faculty of Medicine, Horus University, New Damietta, Egypt; ^5^Internal Medicine Department, Faculty of Medicine, Mansoura University, Mansoura, Egypt; ^6^Pharmacology and Toxicology Department, Faculty of Pharmacy, Tanta University, Tanta, Egypt; ^7^Pharmaceutical Chemistry Department, College of Pharmacy, King Saud University, Riyadh, Saudi Arabia

**Keywords:** atorvastatin, calprotectin, oxidative stress, S1P, ulcerative colitis

## Abstract

**Background:**

Ulcerative colitis (UC) is a chronic inflammatory disorder of the colon. Several preclinical studies investigated the beneficial effects of atorvastatin in colitis. Activation of sphingosine 1 phosphate (S1P)/ tumor necrosis factor-alpha (TNF-*α*)/ interleukin-6 (IL-6) pathways has been confirmed in the pathogenesis of UC and preclinical studies proved the efficacy of atorvastatin on these pathways.

**Aim:**

To investigate the role of atorvastatin on S1P/TNF-*α*/IL-6 pathway in UC.

**Methods:**

Patients with mild to moderate UC were allocated into two groups in this pilot study. For 6 months, Group 1 (placebo group) received both a placebo and 1 g of mesalamine three times daily (t.i.d.). Group 2, (the atorvastatin group) received atorvastatin 80 mg once daily and 1 g of mesalamine t.i.d. A gastroenterologist evaluated the patients’ colitis severity by partial Mayo score index (PMS). Serum IL-6, S1P, TNF-*α*, nitric oxide (NO), erythrocyte sedimentation rate (ESR), C-reactive protein (CRP), and fecal calprotectin were measured before and after treatment. Short Form 36 questionnaire (SF-36) was also assessed. A clinical response was defined as a decline in the rectal bleeding sub score of at least one point, and a decrease in PMS of at least two points. Clinical remission was defined as a PMS of less than 2 and the absence of any single sub score greater than 1.

**Primary outcome:**

Decreased PMS and improved quality of life.

**Secondary outcome:**

Change in the level of measured biomarkers.

**Results:**

Compared to the placebo group (*n* = 24), the atorvastatin group (*n* = 23) exhibited a significant decrease in the level of IL-6 (*p* = 0.001), S1P (*p* = 0.0001), TNF-*α* (*p* = 0.003), NO (*p* = 0.0001), CRP (*p* = 0.015), ESR (*p* = 0.012), PMS (*p* = 0.013), and fecal calprotectin (*p* = 0.0003), and improved SF-36 (*p* = 0.006). In placebo group, the response rate was 83.33% (*n* = 20/24) for PMS, and the remission rate was 45.83% (*n* = 11/24). In the atorvastatin group, the response rate was 91.3% (*n* = 21/23), and the remission rate was 60.8% (*n* = 14/23) for PMS.

**Conclusion:**

Atorvastatin could be an adjunctive therapy for patients with UC.

**Clinical trial registration:**

https://www.clinicaltrials.gov/, Identifier NCT05561062.

## 1 Introduction

Inflammatory bowel disease (IBD) is defined as ongoing episodes of gastrointestinal tract (GI) inflammation caused by an inappropriate immune response to gut bacteria. IBD refers to two forms of idiopathic intestinal disease that differ in their location and level of involvement in the gut wall. Ulcerative colitis (UC) causes widespread inflammation of the intestinal mucosa. UC often affects the rectum (proctitis), but it can also spread to the sigmoid (proctosigmoiditis), beyond the sigmoid (distal UC), or across the colon to the cecum (pancolitis) (1).

Crohn’s disease (CD) causes transmural ulceration of any part of the gastrointestinal tract, but it most commonly affects the terminal ileum and colon. Both disorders are divided into three categories based on their severity (mild, moderate, and sever) and location (1). CD is further characterized according to its phenotype, which might be inflammatory, stricturing, or penetrative. Aside from the GI tract, CD and UC have other extraintestinal symptoms. While the illnesses can be recognized in the majority of patients, at least 10% of patients have symptoms so similar that the two disorders cannot be distinguished at first (2). Patchy inflammation, granulomas, fistulas, and deeply penetrating ulcers are the hallmarks of CD. On the other hand, UC only affects the colon and rectum, resulting in continuous, consistent lesions with mucosal inflammation (3).

IBD develops in genetically predisposed people following an improper immune response to intestinal flora. To present, the cause of IBD remains unknown. Many causes have been identified; however, none are present in all patients. The one persistent aspect of CD is a substantial association with smoke. On the other hand, smoking appears to protect against UC (4). The role of nutrition is still debated. The CARD15 gene has been linked to IBD, however due to its polymorphism nature, it is impossible to predict which portion of the GI tract would be impacted. Compared to CD, UC is less influenced by genetics (5).

Extensive inflammation of the rectal mucosa and colon is a hallmark of UC, an idiopathic, long-term inflammatory disease. However, the precise underlying processes of UC remain unclear (6). Furthermore, because of the high risk of colon cancer, new treatments that slow its course and improve outcomes are needed (7). Numerous factors contribute to the pathophysiology of (UC), including immunological problems, genetic predispositions, epithelial barrier defects, and the environment. Increased permeability of the gut and elevated inflammatory mediators in inflamed mucosa are linked to IBD (8, 9).

Furthermore, mast cells, endothelial cells, and macrophages release interleukin 6 (IL-6), a significant pro-inflammatory cytokine. Studies have indicated that it is crucial for immunological responses, apoptosis, and cell division. In fact, there is clear evidence of a substantial relationship between IL-6, colorectal cancer, and the clinical activity (9). Additionally, research conducted on animal studies have demonstrated the tumor-boosting function of IL-6 during the progression of colitis-associated cancer (CAC) (10). The transcription factor signal transducer and activator of transcription factor 3 (STAT3) plays a fundamental role in mediating the tumor-promoting effect of IL-6, and the IL-6/STAT3 trans-signaling pathway is a key regulator of tumor differentiation (11). Research indicates that IL-6 signaling controls intestinal epithelial cell survival and proliferation and is crucial to the etiology of colorectal cancer and IBD (12).

The breakdown of sphingolipids in the plasma membrane results in the production of sphingosine 1-phosphate (S1P), which is a potent bioactive lipid. It acts as a signaling molecule and performs several metabolic processes by interacting with extracellular receptors and intracellular targets (13). S1P and its metabolites play a role in cell proliferation, apoptosis, and migration, as well as in other disorders such as cancer and inflammation. Within cells, S1P is dephosphorylated by S1P phosphatases to generating sphingosine (14). In mammals, two homologous genes, sphingosine phosphate phosphatase 1 (Sgpp1) and sphingosine phosphate phosphatase 2, encode S1P phosphatases in mammals (15). Sphingosine phosphate lipid phosphatases exhibit a high specificity for sphingoid base phosphates. A family of enzymes known as sphingosine kinases, including sphingosine kinase-1 (SphK1), phosphorylates sphingosine to S1P. These enzymes play critical roles in controlling sphingolipid metabolism (15). SphK1 possesses pro-survival and pro-oncogenic properties and is found in numerous tissues including the heart, brain, and colon. Several studies confirmed the role of S1P in the progression of UC (16, 17).

Oxidative stress is a crucial immunoregulatory component that plays a major role in the development of diseases and may arise subsequent to inflammation (18). There is strong evidence linking the increased production of reactive oxygen and nitrogen species (ROS/RNS) to chronic inflammation in the gut (19). It has been shown that oxidative stress and redox regulation by antioxidants such as catalase, reduced glutathione (GSH), and superoxide dismutase (SOD) play an essential role in the pathogenesis of UC in both animal studies and humans as well. This is because of the overproduction of ROS/RNS (17, 20).

There are several inflammatory markers involved in UC that are considered markers of systemic inflammation in attacks of UC. C- reactive protein (CRP) and erythrocyte sedimentation rate (ESR) are two important inflammatory markers that are elevated in patients with UC (21). The use of ESR to measure systemic inflammation in UC patients has decreased over time as CRP has replaced ESR (22). The latter, which is less impacted by outside factors, is a particular indicator of hepatic inflammation. Additionally, the CRP has a 19-h half-life, making it more sensitive to changes in the inflammatory burden. As a result, it can be utilized to efficiently track therapy response throughout the hospitalization period (22).

In addition to their primary lipid-lowering actions, statins have several pleiotropic effects such as anti-inflammatory and antioxidant effects, improved endothelial function, and immunomodulation (23). Research indicates that in an animal model of dextran sulfate sodium-induced colitis in mice, the severity of intestinal inflammation is reduced by statins (23, 24). In addition, several investigations have revealed the immunomodulatory action of atorvastatin in animal models of induced colitis (25–27). El-Mahdy et al. reported that the combination of atorvastatin and mesalamine significantly reduced IL-6, S1P, and TNF-*α* levels and upregulated tight junction proteins in oxazolone-induced colitis (26). Also, mesalamine combination with metformin and pentoxifylline significantly reduced inflammation and alleviated symptoms in patients with mild and moderate UC (28, 29).

Based on these data, the current study aimed to determine whether the combination of mesalamine and atorvastatin could slow the course of ulcerative colitis and investigate the potential mechanisms underlying these advantageous effects.

## 2 Patients and methods

This study was conducted between January 2023 and March 2024 and 56 patients who met the inclusion criteria were included in the study. The Institutional Review Board of Mansoura University Faculty of Medicine approved this study and provided authorization code MDP.22.08.107. The Helsinki Declaration and its 1964 revisions adhered to the study design and methodology. It was clear to patients that they could withdraw from the study at any time.

### 2.1 Inclusion criteria

Male and female, were included in the study. Effective contraception and a negative pregnancy test should be performed. The study included patients on mesalamine therapy diagnosed previously by colonoscopy. Patients with mild and moderate UC according to partial Mayo score (PMS) were included in this study. PMS is a non-invasive 9-point score that is used as an outcome measure in clinical trials assessing therapy for UC. A score of 0 or 1 indicates remission, 2–4 indicates mild disease, 5–7 indicates moderate disease, and > 7 indicates severe disease. The study included patients on mesalamine therapy diagnosed previously by colonoscopy.

### 2.2 Exclusion criteria

Patients who received systemic or rectal steroids, immunosuppressive medications, or severed-type UC were excluded. Patients with liver or kidney disease were also excluded to prevent the metabolic consequences of atorvastatin. Individuals with a history of hyperlipidemia, musculoskeletal conditions, colorectal malignancy, or complete or partial colectomy were also excluded. Lastly, pregnant women with a history of allergies to the drugs under study were excluded.

### 2.3 Study design

**Figure 1:**
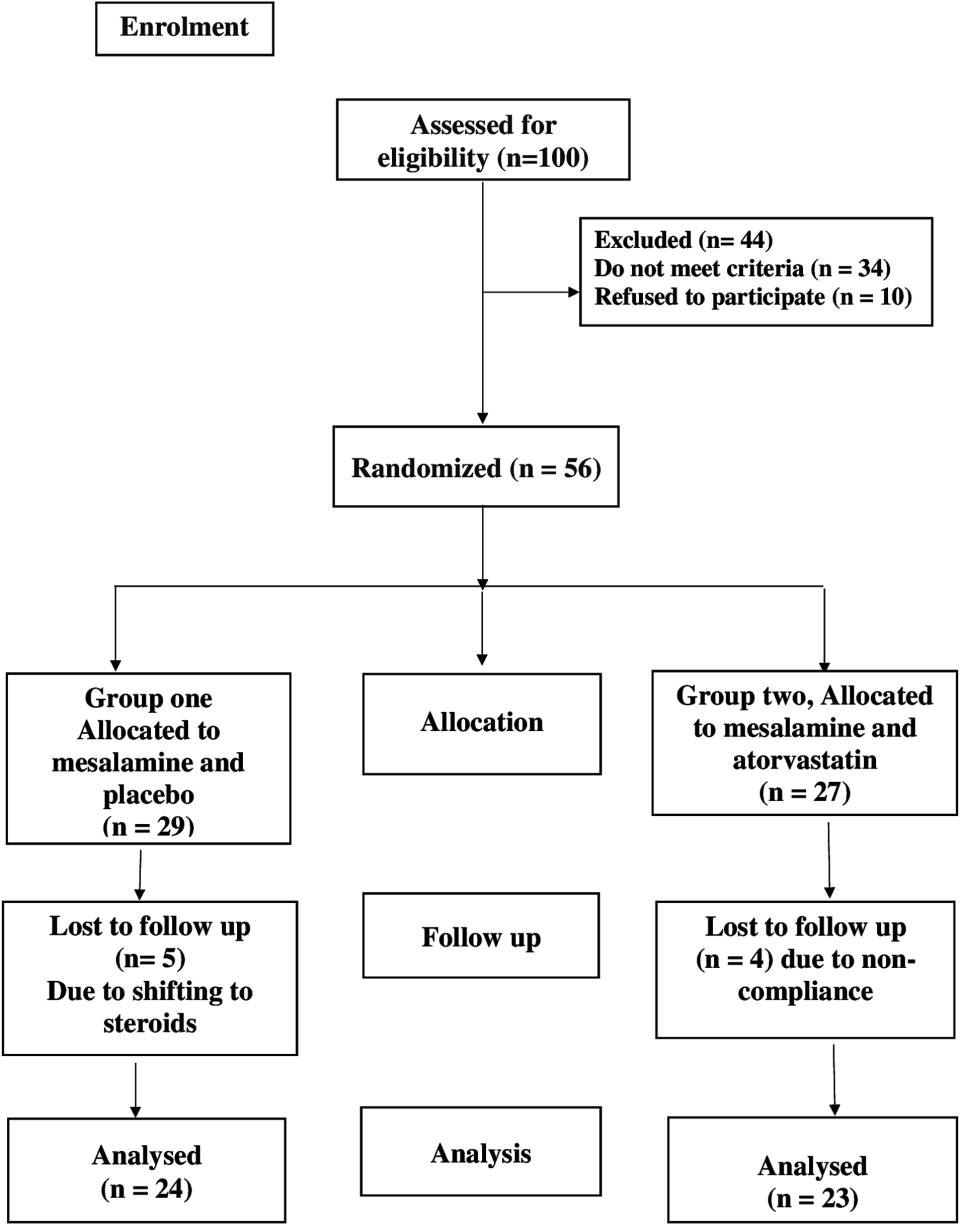
CONSORT diagram showing the flow of participants during the study.

The safety and efficacy of atorvastatin plus mesalamine in the management of UC were assessed in this double-blind randomized controlled clinical study. This trial was registered as NCT05561062 at www.clinicaltrials.gov in 2022. As shown in the CONSORT flow diagram in Figure 1, the patients were randomly divided into two groups. A computer random number generator was used to randomly select permuted blocks for randomization. An unblinded pharmacist provided medication to the patients and was not involved in the outcome. The types of exposure and randomization were hidden from patients and physicians.

Patients who met the requirements for participation and provided written informed consent were split into two groups at random.

Group 1 was the control group; patients received 1 g mesalamine tablets and placebo for 6 months (Pentasa^R^ 500 mg, Multi Pharm, Egypt).

Group 2 received 1 g mesalamine tablets plus 80 mg atorvastatin tablets once daily for 6 months (Ator^R^ 80 mg, EPICO, Egypt).

### 2.4 Sample size calculations

No previous study has determined the actual effect size of atorvastatin treatment on the changes in PMS. This research was intended to be a pilot study according to Julius Sim et al. (30) who recommended a sample size of ≥22 in each group for a small to medium effect size to minimize the combined size. A sample size of 56 patients was randomized in both groups, supposing an *α*-error of 0.05 (2-tailed) and a power of 0.80, and a 20% dropout rate.

### 2.5 Study protocol

In addition to eligibility checks, patients received thorough physical, mental, and psychological assessment. Patients were randomly assigned to receive either placebo and 1 g of mesalamine tablets administered t.i.d. (placebo group), or 1 g of mesalamine tablets taken t.i.d. together with 80 mg of atorvastatin tablets taken once daily (atorvastatin group). The Zeta Pharma Company produced placebo tablets that were identical in appearance to atorvastatin tablets. Along with nutritional and lifestyle counseling, all medications were administered orally to the patients. The selected doses of mesalamine and atorvastatin were 1 g t.i.d (31, 32), respectively, based on previous studies.

### 2.6 Follow-up

Monthly phone calls and meetings were used for follow-up. At the first visit, the patient received a full medical history, liver and kidney function testing, and complete blood counts to rule out any organic abnormalities. Serum biomarkers IL-6, TNF-*α*, S1P, NO, and fecal calprotectin were measured.

### 2.7 Evaluation of colitis

Endoscopy is the gold standard diagnostic tool for UC, but it is an invasive technique, and all patients show noncompliance with this tool (33). Therefore, gastroenterologists replaced the full Mayo score with PMS. Disease severity was determined using PMS. Disease severity was determined using PMS. The PMS is one non-invasive clinical test used to assess the severity of UC. Several studies have documented the use of PMS instead of total mayo score in diagnosis and follow up of UC (33–36). PMS may be used to predict endoscopic and physician Global Assessment scores and serve as a proxy for the full Mayo Score in clinical practice/trials (34). A highly significant correlation was observed between the full Mayo score and PMS (34).

Three subcategories contributed to the composite score: rectal bleeding, stool frequency, and assessment by the general physician. The overall result was in the range 0–9 (33). The PMS results were documented both before and at the end of the study. A clinical response was defined as a decline in the rectal bleeding subscore of at least one point, an absolute score of zero or one, and a decrease in PMS of at least two points and at least 30 percent from the baseline. Clinical remission was defined as a PMS of less than 2 and the absence of any single sub-score greater than 1 (37).

### 2.8 Therapeutic assessments

PMS and SF-36 assessments, as well as measurements of serum IL-6, TNF-*α*, S1P, NO, and fecal calprotectin were used to determine the efficacy of the treatment.

### 2.9 Sample collection

Before the study began and 6 months after the intervention, 10 milliliters of venous blood was drawn from the antecubital vein. The sample was centrifuged for 10 min at 4500 g (Hettich Zentrifugen EBA 20) after the blood was progressively transferred into test tubes and allowed to clot. Two serum aliquots were taken: the first was used for routine tests on the kidney and liver, and the second was frozen at −80°C to measure specific cytokine levels. The stool samples were vortexed, weighed, and dissolved in saline. Clear supernatants were used for calprotectin analysis.

### 2.10 Biochemical analysis

The serum levels of TNF-*α* (catalog no: 201–12-0083), IL-6 (catalog no: 201–12-0091), NO (catalog no: 201–12-1511), calprotectin (catalog no: 201–12-5461), and S1P (catalog no: 201–12-1861) were assessed using commercially available enzyme-linked immunosorbent assay (ELISA) kits, according to the manufacturer’s recommendations. Kits were provided by Sunredio (Shanghai, China). CRP kit (catalog no: DY1707) was supplied by R&D Systems China Co., Ltd.

### 2.11 Statistical analysis

Statistical analyses were performed using Prism version 9 (GraphPad Software Inc., San Diego, California, USA). A continuous variable normal distribution was examined using the Shapiro–Wilk approach. Using the Wilcoxon and Student’s *t*-tests before and after therapy, significant differences were observed between the groups. Before and after therapy, the groups were compared statistically using the Mann–Whitney *U* test and unpaired Student’s *t*-test. While the mean ± SD was used to convey quantitative data, the interquartile range, median, and numbers were used to represent qualitative features. Pearson and Spearman correlation tests were used for parametric and non-parametric data, respectively, to ascertain the correlation between parameters. The McNamar test, Fisher’s exact test, and chi-square test were used for categorical data. Multilinear regression analysis was used to evaluate the effect of age and gender on the primary and secondary outcome’s variable.

### 2.12 Study outcomes

#### 2.12.1 Primary outcomes

Decreased PMS and improved quality of life.

#### 2.12.2 Secondary outcomes

Changes in IL-6, TNF-*α*, S1P, NO, and fecal calprotectin levels.

## 3 Results

### 3.1 Clinical and demographic characteristics

The current study did not find any statistically significant differences between the placebo and atorvastatin groups based on baseline demographic data, including sex (*p* = 0.599), age (*p* = 0.759), body mass index (*p* = 0.242), alanine amino transferase (ALT) (*p* = 0.474), aspartate amino transferase (AST) (*p* = 0.194), hemoglobin (*p* = 0.209), albumin (*p* = 0.479), serum creatinine (SrCr) (*p* = 0.186), total cholesterol (TC) (*p* = 0.411), triglycerides (TG) (*p* = 0.391), low-density lipoprotein (LDL) (*p* = 0.590), and high-density lipoprotein (HDL) (*p* = 0.471) (Table 1). Four patients in the atorvastatin group were lost to follow-up and five patients were withdrawn from the placebo group. Since 47 patients completed the trial, statistical analyses of all measured parameters were performed in accordance with the protocol. Multilinear regression analysis was used to evaluate the effect of age and gender on the primary and secondary outcome’s variable. The results showed that age and gender were not significant predictor for primary and secondary outcomes (Supplementary materials).

**Table 1:** Clinical, demographic and laboratory data of the patients.

Parameter	Placebo group (*n* = 29)	Atorvastatin group (*n* = 27)	*p* value
Age (years)	44.14 ± 14.31	45.22 ± 11.77	0.759
Sex (M/F)	16 /13	13 /14	0.599
BMI (kg/m^2^)	23.21 ± 1.571	23.65 ± 1.142	0.242
Serum ALT (IU/L)	44.59 ± 11.58	46.67 ± 9.872	0.474
Serum AST (IU/L)	42.86 ± 10.75	47.15 ± 13.61	0.194
Hgb (g/dl)	11.73 ± 1.011	12.16 ± 1.450	0.204
Albumin (g/dl)	4.004 ± 0.679	4.140 ± 0.747	0.479
Disease durations (year)	1.6 (0.9–2.6)	1.8 (0.9–2.7)	0.445
SrCr (mg/dl)	0.877 ± 0.193	0.816 ± 0.144	0.186
TC (mg/dl)	162.4 ± 14.48	165.5 ± 13.46	0.411
TG (mg/dl)	133.1 ± 11.37	130.5 ± 11.61	0.391
LDL (mg/dl)	92.23 ± 15.59	94.42 ± 14.59	0.590
HDL (mg/dl)	43.52 ± 6.9	44.96 ± 8.022	0.471
Smoking (*n*)	6	4	0.566

Data are presented as mean ± SD, numbers and interquartile range; placebo group, UC patients treated with mesalamine and placebo; atorvastatin group, UC patients treated with mesalamine plus Atorvastatin, M, Male; F, Female; ALT, alanine aminotransferase; AST, aspartate aminotransferase; Hgb, hemoglobin; Sr, Cr, serum creatinine; TC, total cholesterol; TG, triglycerides; HDL, high-density lipoprotein; LDL, low-density lipoprotein. Statistical significance was set at (*p* < 0.05).

### 3.2 Effect of study drugs on clinical and laboratory data

The Mann–Whitney test and unpaired *t*-test revealed no statistically significant differences between the baseline values of PMS between the two groups (*p* > 0.05).

**Figure 2:**
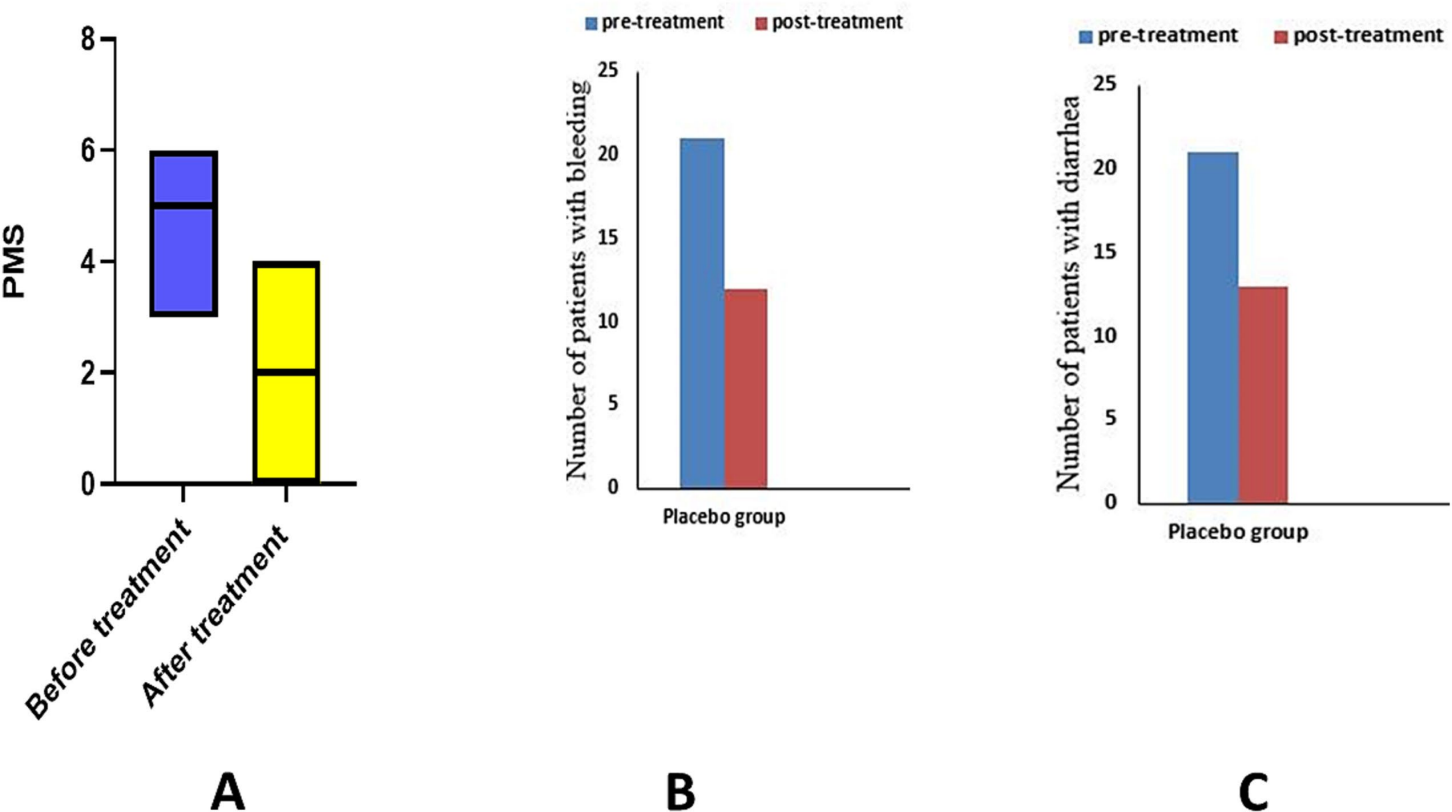
Analysis of PMS and its sub-items in placebo group. **(A)** partial Mayo score, **(B)** number of patients with bleeding, **(C)** number of patients with diarrhea.

After treatment, when comparing the placebo group’s median to the baseline, the Wilcoxon test revealed a significant decrease in PMS (*p* = 0.0001) (Figure 2). Paired *t*-test showed a significant improvement in the mean of SF-36 score (*p* = 0.0001) (Table 2).

**Table 2:** Effect of study medications clinical outcomes.

Character	Placebo group (*n* = 24)			Atorvastatin group (*n* = 23)			*p* value
	Before treatment	After treatment	*p* value	Before treatment	After treatment	*p* value	After treatment
PMS	5 (5–6)	2 (1–2.75)	0.0001^*^	5 (4–6)	1 (0–2)	0.0001^*^	0.013^**^
Rectal bleeding (N)	21/24	12/24	0.014^#^	20/23	4/23	0.0001^#^	0.018^##^
Diarrhea (N)	21/24	13/24	0.030^#^	19/23	6/23	0.0001^#^	0.04^##^
SF-36	33.96 ± 3.290	54.88 ± 9.715	0.0001^a^	34.04 ± 4.301	64.65 ± 13.59	0.0001^a^	0.006^b^

Data are presented as mean and standard deviation, numbers, median and interquartile range, placebo group, UC patients treated with mesalamine and placebo, atorvastatin group, and UC patients treated with mesalamine plus atorvastatin. PMS, partial Mayo score, SF-36, short form 36 questionnaire. (^*^) Level of significance within the same group using the Wilcoxon test. (^**^) Level of significance between groups using the Mann–Whitney test. (^#^) Level of significance within the same group using McNamar’s test. (^##^) Level of significance between the groups using the chi-square test.

a Level of significance within the same group using paired *t*-test.

b Level of significance between groups using unpaired *t*-test. Statistical significance was set at (*p* < 0.05).

**Figure 3:**
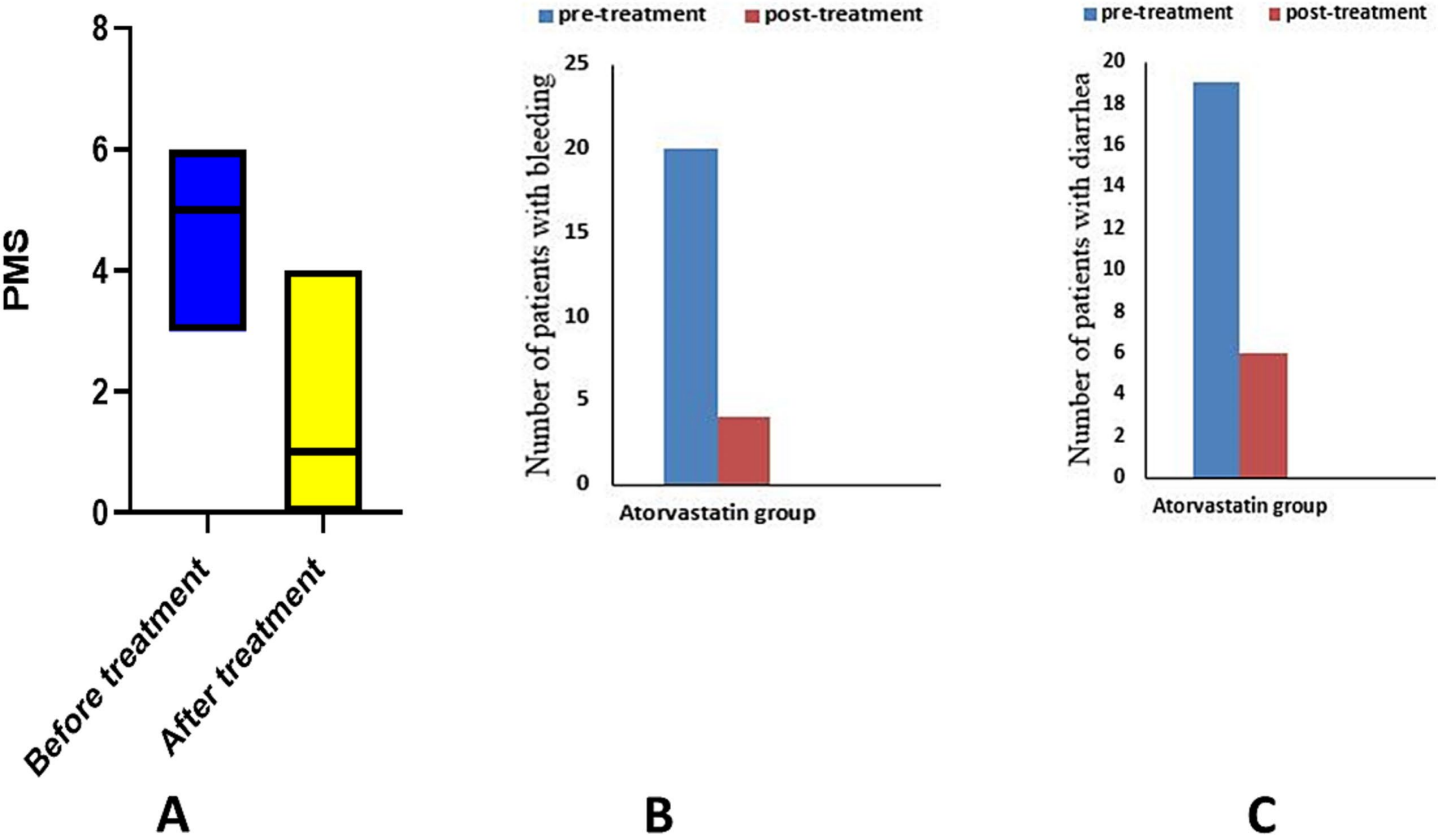
Analysis of PMS and its sub-items in atorvastatin group. **(A)** partial Mayo score, **(B)** number of patients with bleeding, **(C)** number of patients with diarrhea.

After treatment, when comparing the median atorvastatin group to the baseline, the Wilcoxon test revealed a significant decrease in PMS (*p* = 0.0001) (Figure 3), and paired *t*-test showed a significant improvement in the mean of SF-36 score (*p* = 0.0001) (Table 2).

Between group comparison, Mann–Whitney test demonstrated a statistically significant decrease in the median PMS after 6 months of intervention (*p* = 0.013), and the unpaired *t*-test revealed a significant increase in the mean SF-36 score (*p* = 0.006) (Table 2).

Using the McNamar test, the placebo group showed a significant decrease in the number of patients with bleeding (*p* = 0.014) and diarrhea (*p* = 0.03). The atorvastatin group showed a significant decrease in the number of patients with bleeding (*p* = 0.0001) and diarrhea (*p* = 0.0001) in the McNamar test (Table 2).

The chi-square test revealed a significant difference in the number of patients with diarrhea (*p* = 0.04) and bleeding (*p* = 0.018) between the two studied groups.

The response and remission rates in the placebo group were 83.33% (20/24) and 45.83% (11/24) respectively, for PMS. In the atorvastatin group, the response rate was 91.3% (*n* = 21/23) and the remission rate was 60.8% (*n* = 14/23) for PMS.

### 3.3 Effect of study medications on serum biomarkers

**Figure 4:**
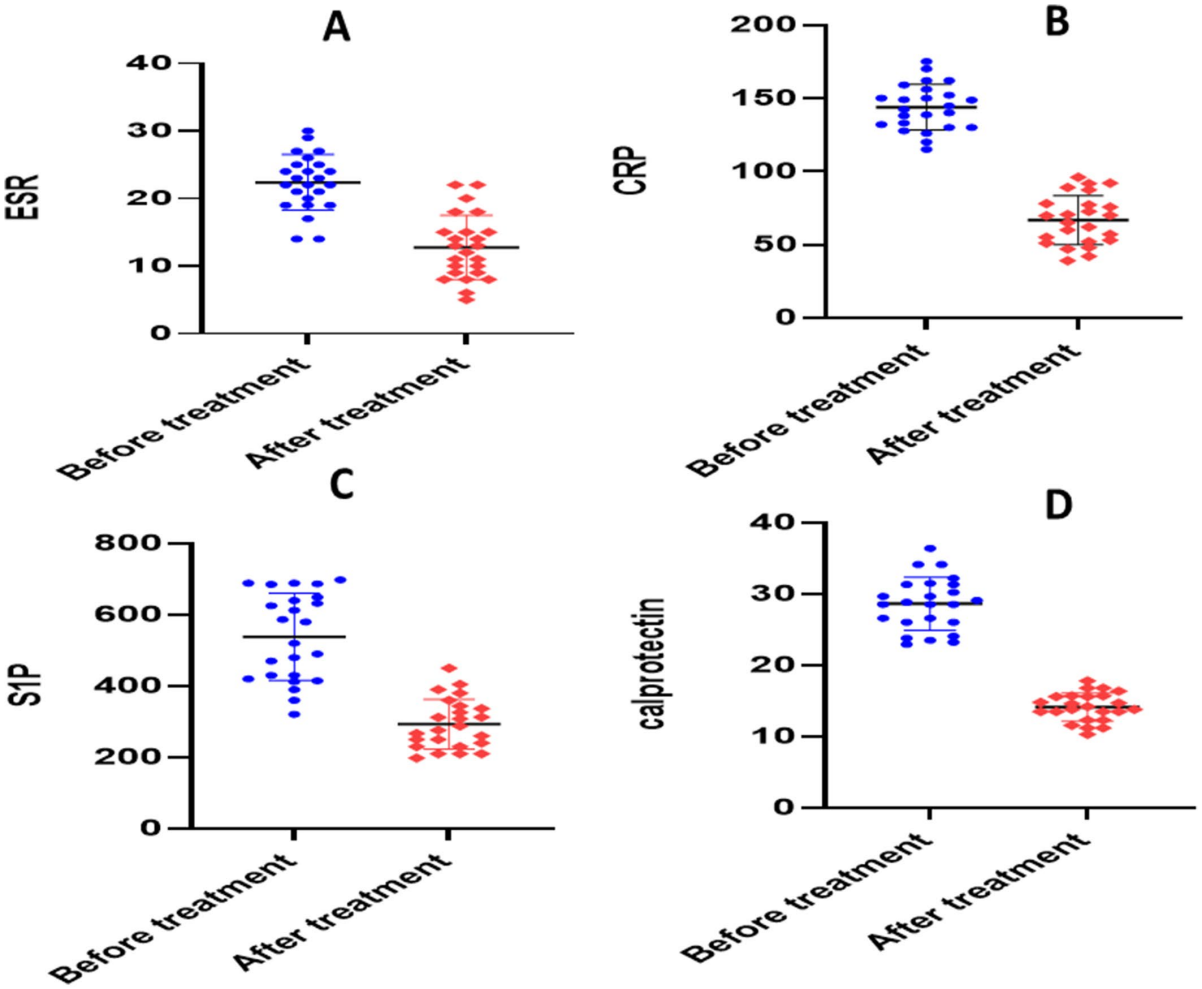
Analysis of biological markers in placebo group. **(A)** Erythrocyte sedimentation rate (ESR), **(B)** C- reactive protein (CRP), **(C)** calprotectin, **(D)** sphingosine 1 phosphate.

**Figure 5:**
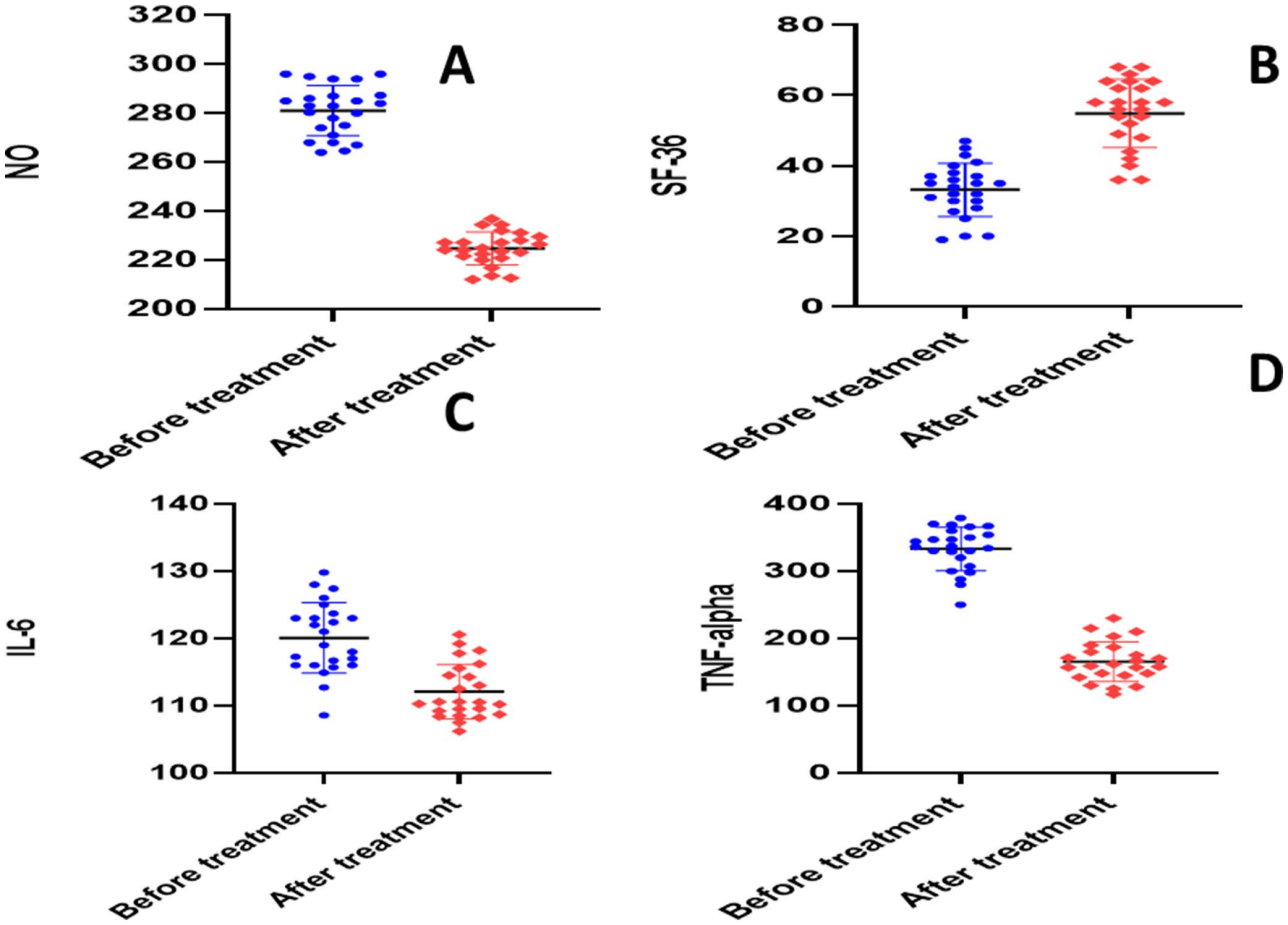
Analysis of biological markers in placebo group. **(A)** Nitric oxide (NO), **(B)** short form-36 questionnaire (SF-36), **(C)** interleukin 6 (IL-6), **(D)** tumor necrosis factor alpha (TNF-alpha).

There was no statistically significant difference in baseline values between the two groups according to an unpaired *t*-test (*p* > 0.05). The paired *t*-test for the placebo group showed significant reductions in each of the following parameters when compared to the baseline: calprotectin (28.64 ± 3.704 versus 14.13 ± 1.97, *p* = 0.0001), TNF-*α* (336.2 ± 20.88 versus 158.6 ± 9.56, *p* = 0.0001), NO (281.1 ± 10.26 versus 224.7 ± 6.705, *p* = 0.0001), S1P (656 ± 30.46 versus 306.5 ± 23.17, *p* = 0.0001), ESR (22.38 ± 4.13 versus 12.21 ± 2.91, *p* < 0.0001), CRP (140.6 ± 8.22 versus 71.18 ± 8.27, *p* < 0.0001), and IL-6 (120.1 ± 5.254 versus 112.1 ± 4.052, *p* = 0.0001) (Table 3 and Figures 4, 5).

**Table 3:** Effect of study medications on serum and fecal parameters.

	Placebo group (*n* = 24)			Atorvastatin group (*n* = 23)			*p* value
Character	Before treatment	After treatment	*p* value	Before treatment	After treatment	*p* value	After treatment
IL-6 (pg/ml)	120.1 ± 5.254	112.1 ± 4.052	0.0001^*^	119.8 ± 4.208	107 ± 6.017	0.0001^*^	0.001^**^
TNF-α (pg/ml)	336.2 ± 20.88	158.6 ± 9.56	0.0001^*^	340.9 ± 13.93	141.1 ± 25.76	0.0001^*^	0.003^**^
Fecal calprotectin (ng/ml)	28.64 ± 3.704	14.13 ± 1.97	0.0001^*^	30.45 ± 6.596	11.37 ± 2.78	0.0001^*^	0.0003^**^
S1P (pg/ml)	656 ± 30.46	306.5 ± 23.17	0.0001^*^	651.4 ± 22.59	199.7 ± 32.85	0.0001^*^	0.0001^**^
NO (μmol/L)	281.1 ± 10.26	224.7 ± 6.70	0.0001^*^	278.1 ± 10.20	178.6 ± 36.65	0.0001^*^	0.0001^**^
ESR (mm/h)	22.38 ± 4.13	12.21 ± 2.91	<0.0001^*^	23.04 ± 3.77	9.870 ± 3.09	<0.0001^*^	0.012^**^
CRP (pg/ml)	140.6 ± 8.22	71.18 ± 8.27	<0.0001^*^	141.0 ± 5.478	64.08 ± 10.84	<0.0001^*^	0.015^**^

Data are presented as mean ± SD, Placebo group, UC patients treated with mesalamine and placebo, atorvastatin group, UC patients treated with mesalamine plus atorvastatin, IL-6, interleukin 6, TNF-α, tumor necrosis factor-alpha, S1P, and sphingosine 1 phosphate, NO, nitric oxide, ESR, erythrocyte sedimentation rate, CRP, C-reactive protein. (^*^) Level of significance within the same group determined using a paired *t*-test. ^(**)^ Level of significance between groups using an unpaired *t*-test. Statistical significance was set at (*p* < 0.05).

**Figure 6:**
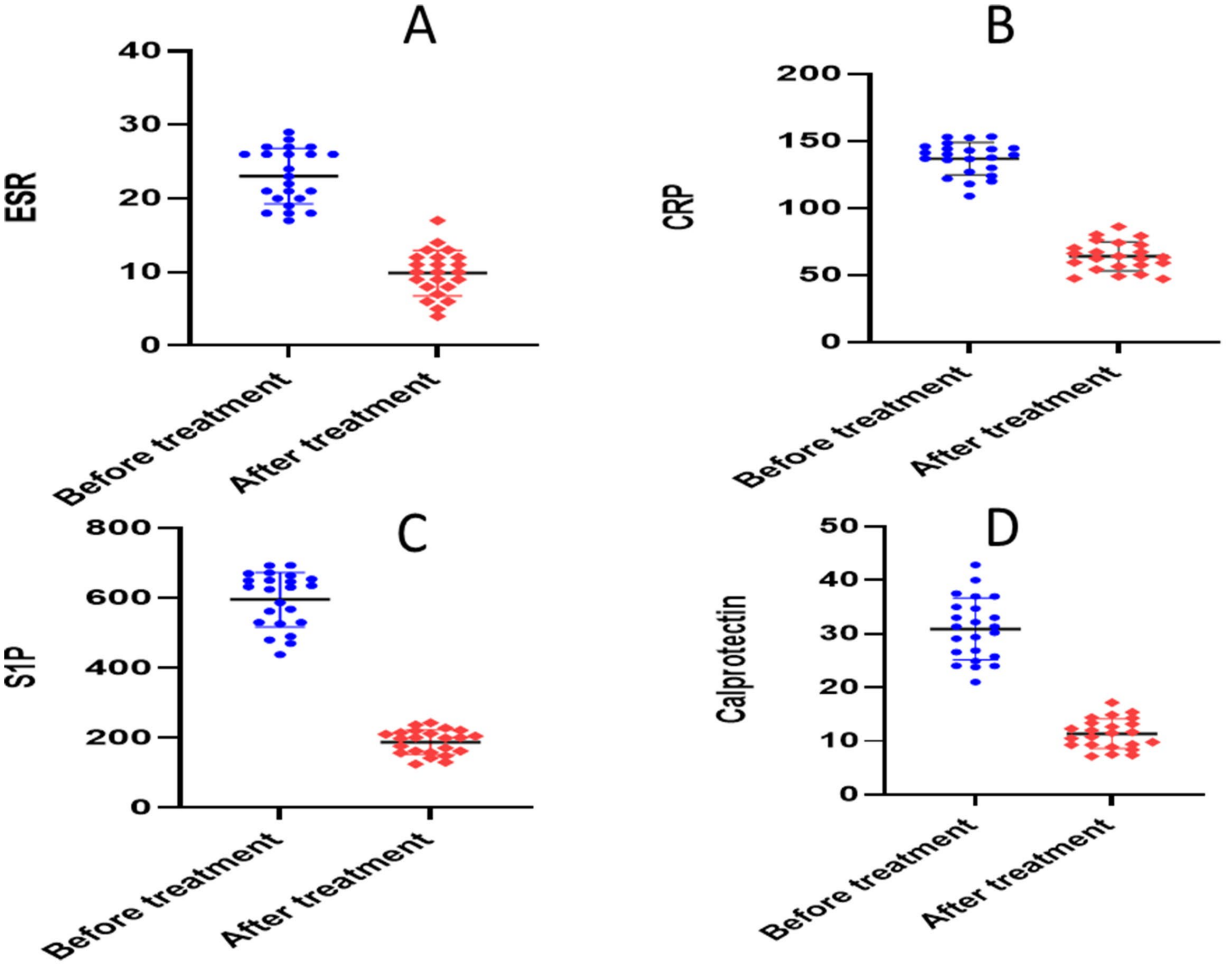
Analysis of biological markers in atorvastatin group. **(A)** Erythrocyte sedimentation rate (ESR), **(B)** C- reactive protein (CRP), **(C)** calprotectin, **(D)** sphingosine 1 phosphate.

**Figure 7:**
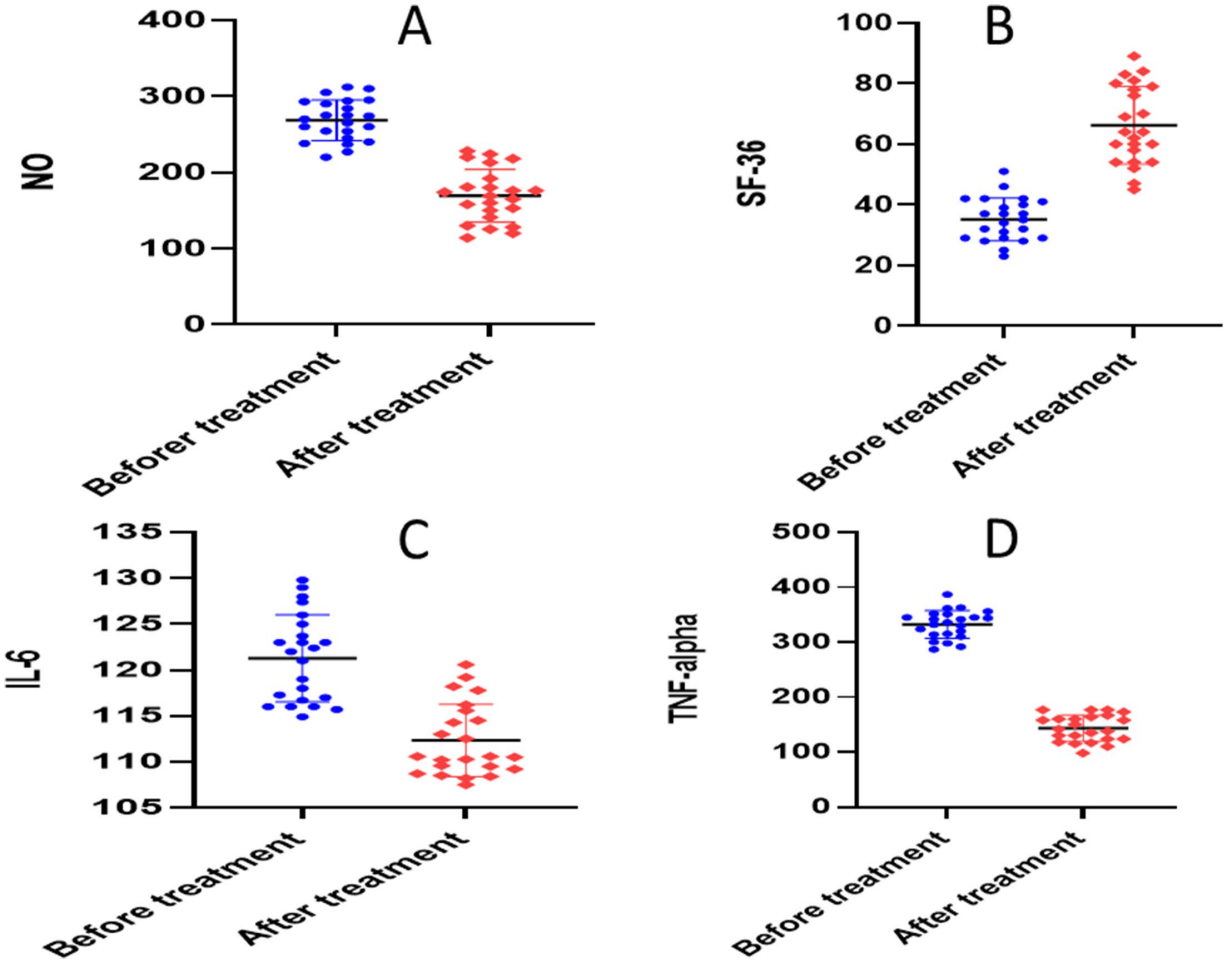
Analysis of biological markers in atorvastatin group. **(A)** Nitric oxide (NO), **(B)** short form-36 questionnaire (SF-36), **(C)** interleukin 6 (IL-6), **(D)** tumor necrosis factor alpha (TNF-alpha).

The paired *t*-test for the atorvastatin group showed significant reductions in each of the following parameters when compared to the baseline: calprotectin (30.45 ± 6.596 versus 11.37 ± 2.78, *p* = 0.0001), TNF-*α* (340.9 ± 13.93 versus 141.1 ± 25.76, *p* = 0.0001), NO (278.1 ± 10.20 versus 178.6 ± 36.65, *p* = 0.0001), S1P (651.4 ± 22.59 versus 199.7 ± 32.85, *p* = 0.0001), ESR (23.04 ± 3.77 versus 9.870 ± 3.09, *p* < 0.0001), CRP (141.0 ± 5.478 versus 64.08 ± 10.84, *p* < 0.0001), and IL-6 (119.8 ± 4.208 versus 107 ± 6.017, *p* = 0.0001) (Table 3 and Figures 6, 7).

Between group comparison, after 6 months of intervention, the unpaired *t*-test showed statistically significant differences in all studied markers: calprotectin (*p* = 0.0003), NO (*p* = 00001), S1P (*p* = 00001), TNF-α (*p* = 0.003), ESR (*p* = 0.012), CRP (*p* = 0.015), and IL-6 (*p* = 0.001) (Table 3).

### 3.4 Analysis of drug-related adverse effects between the groups

Table 4 shows that there were no statistically significant differences in the following side effects between the two groups: muscle weakness (*p* = 0.196), flatulence (*p* = 0.461), headache (*p* = 0.999), dizziness (*p* = 0.999), skin rash (*p* = 0.999), vomiting (*p* = 0.7), and stomach pain (*p* = 0.524).

**Table 4:** Analysis of drug related side effects between the studied groups.

Side effect	Placebo group (*n* = 24)	Atorvastatin group (*n* = 23)	*p* value
Muscle weakness	2	5	0.196
Vomiting	3	4	0.700
Skin rash	3	3	0.999
Stomach pain	6	4	0.524
Headache	3	2	0.999
Flatulence	3	5	0.461
Dizziness	4	4	0.999

Data are presented as numbers, Placebo group, UC patients treated with mesalamine and placebo, atorvastatin group, UC patients treated with mesalamine plus Atorvastatin, significance (*p* < 0.05) was determined using the chi-square test and Fisher’s exact test.

### 3.5 Correlation analysis between the studied biomarkers

Spearman’s correlation test revealed a significant positive correlation between PMS and calprotectin (*p* < 0.0001, *r* = 0.7) and a significant negative correlation between PMS and SF-36 (*p* < 0.0001, *r* = − 0.6). The Pearson correlation test showed a significant negative correlation between SF-36 and calprotectin levels (*p* < 0.0001, *r* = − 0.76).

## 4 Discussion

Prolonged and recurrent inflammation of the digestive tract is the hallmark of ulcerative colitis (UC). Since it is linked to an increased probability of developing colon cancer, it is critical to continuously discover novel treatments to slow the disease course, induce remission, and prevent relapse to improve clinical outcomes (38). Pharmacological treatments to reduce inflammation and, in extreme situations, surgery are common treatment approaches (39). Aminosalicylates, corticosteroids, and immunomodulators (methotrexate, thiopurines, calcineurin inhibitors, and Janus kinase inhibitors) are examples of small compounds (39). Biological medications include ustekinumab against interleukin-12/23, vedolizumab against integrin *α*4β7, and antibodies infliximab, golimumab, adalimumab, and certolizumab pegol against TNF-α (40). Most of used drugs have adverse effects, so there is a continuous need for new therapeutic approaches.

Randomized clinical research found that atorvastatin did not appear to have any positive effects on acute UC exacerbations, and in some patients, there was a paradoxical increase in disease severity (41). Some patients in this study experienced an increase in disease activity after taking atorvastatin, but not all of the patients were included in the study; objective measures of disease activity, such as histology, endoscopic examination, and biochemical parameters of inflammation, did not support these findings. Numerous studies have indicated that atorvastatin administered at a high dose of 80 mg/day is superior to that administered at a low dose in terms of lowering inflammation. Notably, the high-dose atorvastatin group had a noticeable decrease in inflammatory markers (42, 43). Furthermore, Grip et al. revealed that atorvastatin (80 mg daily) affected clinical symptoms and inflammatory indicators in CD (44). Therefore, the administration of a small dose of atorvastatin (20 mg/day) and the short study duration (only 2 months) may have contributed to the lack of effectiveness of atorvastatin in the above study. These factors may not have been sufficient to achieve anti-inflammatory properties and attenuate the aggravation of UC.

To our knowledge, this is the first clinical pilot study to investigate the effects of atorvastatin on the IL-6/TNF-*α*/S1P pathway in patients with UC. Other studies have validated the efficacy of atorvastatin in UC treatment (45, 46). Additionally, retrospective cohort research has shown that among patients with IBD, statin administration is linked to a decrease in steroid use (47). Compared with the placebo group, the atorvastatin group showed a significant improvement in health-related quality of life and a significant reduction in PMS, response, and remission rates. This result is likely attributable to the combination of mesalamine and atorvastatin, both of which have anti-inflammatory properties that help relieve gastrointestinal pain and restore normal functioning. These results are consistent with those of other studies (26, 48, 49). Our findings support the notion that atorvastatin may alleviate symptoms in patients with mild-to-moderate UC. Administration of atorvastatin to experimental colitis resulted in a considerable decrease in the colonic endoscopic score and alleviated histological and immunohistochemical alterations when compared to the same parameters before treatment (26). The use of PMS in predicting response and relapse in patients with UC is highly encouraged, as it is a non-invasive tool, and many patients showed good prognosis and compliance in contrast to the full Mayo score, which requires anesthesia and surgery. Clinical studies have revealed that endoscopic findings are highly correlated with PMS, and that PMS can replace endoscopy in the diagnosis of UC (50). The authors reported that PMS was closely correlated with the total Mayo scores at weeks 4 and 8. The model to predict total PMS showed excellent correlation and good agreement with the total Mayo score at weeks 4 and 8 and accurately classified disease severity (50).

As there were no discernible differences in the demographic or clinical variables across the groups at the start of the investigation, the study medications were primarily responsible for the therapeutic results. In the current investigation, the placebo group showed an improvement in SF-36 and a substantial decrease in serum IL-6, TNF-*α*, SIP, NO, and fecal calprotectin levels compared to the baseline value. In addition, the PMS in this group’s significantly lower than the baseline value. These observations were undoubtedly caused by mesalamine, which is widely used to treat mild to moderate UC (51). These results are in line with those of previous studies that investigated the effects of mesalamine on NO, TNF-α, S1P, and IL-6 in animal models of colitis (26, 27).

In this study, we showed that mesalamine combined with atorvastatin may slow the progression of UC by lowering oxidative stress markers such as NO and targeting IL-6/TNF-α/S1P signaling. Cytokine profiling is a reliable and non-invasive method for predicting the therapeutic success of biological medications in patients with IBD. Of the cytokines investigated, IL-6 was found to have a substantial correlation with the duration of the disease and established indicators of inflammation, such as fecal calprotectin and CRP, and varied significantly throughout biological treatment (52). Prior clinical trials with infliximab-treated patients demonstrated that responders’ plasma IL-6 levels were considerably lower than those of non-responders at baseline and after 8 weeks of treatment (53, 54). Surprisingly, at 12 months of biologic therapy, the decline in IL-6 levels from baseline to week 10 of therapy was thought to be an independent predictor of clinical response (55). The results of the current investigation showed that mesalamine administration considerably reduced IL-6 levels. These findings are consistent with those of previous studies (56, 57). Additionally, atorvastatin and mesalamine significantly reduced IL-6 signaling, consistent with previous data (26, 58).

In comparison with both placebo groups in our study, the TNF-*α* level in the atorvastatin group was considerably lower. These results are consistent with those of previous studies (26, 58, 59). The suppression of inflammatory markers and peroxisome proliferator–activated receptor (PPAR)-gamma-dependent mechanism may be responsible for the anti-inflammatory actions (60). According to Liu et al., atorvastatin induces receptor expressed on myeloid cells-1 expression, decreases nuclear factor kappa-light-chain-enhancer of activated B cells (NF-jB) p65 expression, regulates tissue transglutaminase expression, and increases nuclear factor erythroid 2-related factor (Nrf-2) expression (61). However, Hussein et al. revealed that atorvastatin therapy significantly increases the levels of inflammatory cytokines (62). Whether atorvastatin stimulates pro-inflammatory cytokine release in mice infected with *E. coli* is currently being studied. In this study, TNF-α, and IL-6 levels were dramatically reduced by atorvastatin, but not in the presence of an *E. coli* component. One possibility is that the existence or lack of an immune response influences how statins interact with an organism at the cellular and molecular levels (62).

Our findings demonstrated that compared to both the placebo group and its baseline, the atorvastatin group had significantly decreased NO levels. These findings are consistent with previous publications (26, 63). El-Mahdy et al. observed that the combination of atorvastatin and mesalamine significantly mitigated oxazolone-induced oxidative damage, as shown by a considerable increase in colonic GSH and a substantial reduction in NO content; these findings are consistent with earlier observations (26). Adenosine monophosphate-activated protein kinase (AMPK) activation contributes to mesalamine-mediated beneficial antioxidant effects by interfering with the proinflammatory NF-κB cascade (64). By decreasing malondialdehyde and promoting the production of antioxidant enzymes such as SOD, atorvastatin can significantly decrease oxidative stress (48, 65). Sasaki et al. reported that mice lacking endothelial nitric oxide synthase (eNOS) in induced colitis showed increased disease progression (48).

Polymorphonuclear neutrophils migrate from circulation to the intestinal mucosa during active intestinal inflammation. Any disruption of the mucosal architecture caused by the inflammatory process causes neutrophils to leak into the lumen, where they release calprotectin, which is then excreted in stool (66). There was a good correlation between the severity of UC and the amount of calprotectin present in the stools (67). The current study demonstrated that atorvastatin in combination with mesalamine significantly reduced calprotectin levels, CRP, and ESR compared with mesalamine alone. Our results are in line with another study (68). Grip and Olof demonstrated a significant correlation between calprotectin levels and inflammatory chemokines in (CD) patients (68). Additionally, they demonstrated that high doses of atorvastatin lowered the clinical disease activity and plasma levels of CRP in patients with CD. The amount of fecal calprotectin in a patient’s stool indicates mucosal healing in patients with UC and corresponds with endoscopic and histologic inflammation (69). Statin inhibits the inflammatory response by lowering the levels of CRP and ESR (70).

There was a strong correlation between fecal calprotectin and PMS and a strong negative correlation between fecal calprotectin levels and quality of life. These observations suggest that reduction in calprotectin levels leads to attenuation of UC and reduced disease activity in patients with UC. The reduction in disease activity and fecal calprotectin levels leads to an improvement in the patients’ quality of life. Several studies demonstrate that fecal calprotectin was strongly correlated with disease activity in patients with IBDs (71, 72).

Our study did not include colonoscopy or histological assessment, which is a limitation of our study, because we relied on PMS and other markers to measure response and remission. There is a continuous need for new and better diagnostic tools for UC. Since the patients refuse colonoscopy due to its invasive nature. Different approaches would be beneficial in the diagnosis of UC. One interesting approach is smart bionanomaterials (73). The use of smart bionanomaterials has produced encouraging results in this regard, protecting the active component and making them helpful tools in the treatment of IBD (73). Smart bionanomaterials for IBD therapy and diagnostics are mostly created through crosslinking (hydrogels), electrospinning (nanofibers), dialysis and emulsification (nanoparticles), and molecular cloning (smart probiotics). The materials often respond to pH, temperature, ROS, particular biomolecules, and magnetic fields (74). The inflammatory microenvironment in IBD is also used to stimulate the release of medications from smart bionanomaterials in the inflamed bowel (74). Smart bionanomaterials were stimulated with IBD-specific molecules, including esterases, matrix metalloproteinases, CD98, Ly6C, CD44, TNF-*α*, and folate receptors, to release active agents (74).

## 5 Conclusion

This is the first clinical study to examine the effects of atorvastatin on IL-6/TNF-α/S1P in patients with UC. These biomarkers could be valuable for understanding the protective mechanism of atorvastatin in UC. For those with UC, atorvastatin may be an effective added treatment. The combination of atorvastatin and mesalamine may have significant anti-inflammatory and antioxidant effects by reducing TNF-α, IL-6, S1P, and fecal calprotectin levels.

Despite the limitations of the study (short follow-up time, inadequate sample size, and lack of varied doses), we encourage larger-scale randomized clinical trials with larger sample sizes and longer durations to validate our findings. Healthy controls may be compared with patients with UC. In Egypt, there are legal restrictions on enrolling healthy volunteers for research trials. It would have been advisable to evaluate creatine kinase enzyme and lipid profiles after treatment.

## Data Availability

The raw data supporting the conclusions of this article will be made available by the authors, without undue reservation.
