# Supplementary material for: Effect of a high dose atorvastatin as adjuvant therapy to mesalamine in attenuating inflammation and symptoms in patients with ulcerative colitis: a randomized controlled pilot study
Source: Front Med (Lausanne). 2025 Jan 22;11:1490178. doi: 10.3389/fmed.2024.1490178 (PMC11794804; doi:10.3389/fmed.2024.1490178)
Supplement: Supplementary file 1 [file Data_Sheet_1.pdf]

Multilinear regression analysis was used to evaluate the effect of age and gender on the primary and secondary outcome's variable. The results showed that age and gender were not significant predictor for primary and secondary outcomes according to the following Table.

Multilinear regression analysis for placebo group

Independent variables	β	T	p-value	Dependent variables	Significance
Age	0.011	0.585	0.564	PMS	NS
Gender	0.405	0.743	0.465		
Age	0.077	0.493	0.627	SF-36	NS
Gender	-0.481	0.112	0.911		
Age	-0.047	0.725	0.479	IL-6	NS
Gender	0.248	0.140	0.889		
Age	-0.048	0.316	0.755	TNF	NS
Gender	-3.70	0.894	0.381		
Age	-0.180	1.903	0.070	NO	NS
Gender	-5.542	2.148	0.084		
Age	0.024	0.761	0.454	Calprotectin	NS
Gender	-0.133	0.154	0.879		
Age	0.994	0.284	0.778	S1P	NS
Gender	-17.36	1.854	0.078		
Age	-0.271	2.260	0.098	CRP	NS
Gender	-0.111	0.134	0.973		
Age	0.011	0.250	0.805	ESR	NS
Gender	-1.149	0.909	0.373		

Multilinear regression analysis for atorvastatin group.

Independent variables	β	T	p-value	Dependent variables	Significance
Age	- 0.055	2.822	0.105	PMS	NS
Gender	0.065	0.139	0.890		
Age	- 0.215	0.817	0.423	SF-36	NS
Gender	-3.854	0.612	0.547		
Age	0.169	1.530	0.141	IL-6	NS
Gender	0.559	1.348	0.192		
Age	-0.411	0.974	0.341	TNF	NS
Gender	-5.492	3.036	0.094		
Age	-0.199	0.277	0.784	NO	NS
Gender	-10.33	0.603	0.553		
Age	0.002	0.037	0.970	Calprotectin	NS
Gender	-0.300	0.229	0.820		
Age	-0.763	0.284	0.236	S1P	NS
Gender	-1.090	0.073	0.942		
Age	-0.028	0.140	0.889	CRP	NS
Gender	5.614	1.143	0.266		
Age	0.002	0.047	0.962	ESR	NS
Gender	-0.275	0.198	0.851		
